# Porcelain Teeth

**Published:** 1845-12

**Authors:** 


*Porcelain Teeth.*—We are indebted to Messrs. Wilkinson and Armstrong, of Philadelphia, for some very beautiful specimens of incorruptible teeth. In their appearance, they bear a very close resemblance to the human teeth, both in color and shape; and although we have not had an opportunity of testing their strength, we should think them a good article.

